# The Association Between Missed Nursing Care and Job Satisfaction Among Nurses in Saudi Arabian Hospitals: A Cross-Sectional Study

**DOI:** 10.3390/nursrep15080296

**Published:** 2025-08-12

**Authors:** Bushra Alshammari, Munirah Matar Alshammari, Nadiah A. Baghdadi

**Affiliations:** 1 Medical Surgical Nursing Department, College of Nursing, University of Hail, Hail 2440, Saudi Arabia; 2 Pediatric Emergency Department, Maternity and Children’s Hospital, Hail Health Cluster, Hail 2440, Saudi Arabia; malshammari189@moh.gov.sa; 3 Nursing Management and Education Department, College of Nursing, Princess Nourah bint Abdulrahman University, P.O. Box 84428, Riyadh 11671, Saudi Arabia; nabaghdadi@pnu.edu.sa

**Keywords:** missed nursing care, job satisfaction, nursing workforce, Saudi Arabia, cross-sectional study

## Abstract

**Background/Objectives**: Missed nursing care (MNC), defined as any omitted or delayed aspect of required patient care, is a global concern affecting both patient outcomes and nurse well-being. In Saudi Arabia, few studies have examined its relationship with job satisfaction. This study assessed the prevalence of MNC, job satisfaction, and their association among nurses in government hospitals. **Methods**: A correlational, cross-sectional study was conducted between February and May 2025 in government hospitals across Albahah, Hail, and Almadina. A total of 366 registered nurses participated. Data were collected via a structured online questionnaire including demographic information, the Missed Nursing Care Scale, and the Minnesota Satisfaction Questionnaire (MSQ). Data were analyzed using descriptive statistics, Spearman’s correlation, and regression analysis. **Results**: Basic care activities such as mouth care, bathing, and meal assistance were the most frequently missed tasks, mainly due to staffing shortages and high patient loads. The median MSQ score was 60 (44–71 Interquartile Range), reflecting moderate job satisfaction. A significant negative correlation was observed between MNC and job satisfaction (r = −0.267, *p* < 0.001), indicating that increased missed care was associated with lower satisfaction levels. **Conclusions**: The findings highlight the urgent need for healthcare administrators and policymakers to implement strategies to improve staffing levels and work environments. Addressing these organizational factors is essential to reducing missed nursing care, enhancing nurse job satisfaction, and ultimately improving patient safety and care quality in Saudi government hospitals.

## 1. Introduction

Nursing care delivery remains fundamental to patient outcomes and safety in healthcare systems worldwide. However, the phenomenon of missed nursing care (MNC), defined as any required patient care that is omitted or delayed, has become a critical global issue [1,2,3]. The prevalence of missed nursing care has been reported to range between 30% and 70% globally across various clinical settings, with higher rates observed in high-acuity departments such as emergency, intensive care, and surgical units [1,4,5]. In regions such as the Middle East, including Saudi Arabia, emerging evidence suggests similar or even higher prevalence rates due to systemic challenges like staffing shortages and workforce diversity [6,7]. MNC encompasses clinical, psychological, and administrative tasks and has been associated with excessive workloads, high patient-to-nurse ratios, resource constraints, and organizational inefficiencies [8,9]. The repercussions of MNC extend beyond compromised patient care to include medication errors, patient falls, delayed or incorrect treatments, pressure ulcers, infections, and ultimately higher morbidity and mortality rates [3,10]. These patient safety risks are compounded by the adverse effects on nurses themselves, leading to dissatisfaction, burnout, moral distress, and high turnover intentions, which further weaken the healthcare workforce [4,5,11,12].

A recent meta-analysis has consistently demonstrated a negative association between MNC and job satisfaction, suggesting that nurses who report higher levels of missed care are often less satisfied with their work [4]. In this context, job satisfaction refers to nurses’ overall affective and cognitive evaluation of their work experience, including factors such as workload, professional autonomy, recognition, work environment, and interpersonal relationships [13,14]. It is recognized as a key determinant influencing nurse retention, performance, and ultimately, patient care quality.

This study is conceptually framed within the Missed Nursing Care Model as proposed by Kalisch and Williams [2], which posits that organizational antecedents—such as staffing adequacy, teamwork, leadership, and work environment—affect internal processes that lead to missed care events [15]. The model highlights both the direct impact of these factors on patient outcomes and their indirect influence through nurse-related variables like job satisfaction. Based on this framework, we hypothesize that higher levels of missed nursing care are associated with lower levels of job satisfaction among nurses working in Saudi Arabian government hospitals. By adopting this framework, our study systematically examines how organizational dynamics contribute to both MNC prevalence and nurses’ job satisfaction within the unique cultural and systemic context of Saudi Arabian government hospitals.

Saudi Arabia has one of the most rapidly expanding healthcare systems globally, driven by the ambitious national Vision 2030 plan to improve health outcomes, hospital infrastructure, and workforce development [6,16]. The country provides healthcare services to a highly diverse and growing population, including both Saudi nationals and a large expatriate workforce. Despite these advancements, persistent challenges such as high workloads, inconsistent staffing levels, language barriers, and cultural diversity among healthcare workers continue to affect the delivery of nursing care [7,17]. Such factors can hinder effective communication and teamwork in clinical settings [18,19]. Additionally, traditionally hierarchical organizational structures may limit nurses’ autonomy and voice in the workplace. Taken together, these context-specific challenges—high stress and burnout, staffing constraints, and communication barriers—raise concerns that missed nursing care could be prevalent in Saudi hospitals, potentially even more so than in some Western healthcare systems with more stable nurse staffing. However, there is a paucity of localized research quantifying MNC in Saudi Arabia or examining how it relates to nurse outcomes like job satisfaction. This gap underscores the need to investigate MNC within the Saudi context, where unique cultural and systemic factors might influence both the occurrence of missed care and its impact on nurses and patients [16].

Despite these global insights, limited research has been conducted in the Saudi context, where cultural norms, hierarchical structures, and communication barriers can uniquely influence nursing care practices [1,7]. The lack of localized studies underscores the urgent need to understand the dynamics of MNC and job satisfaction within the Saudi healthcare system to formulate appropriate interventions and policies [20]. Although job satisfaction and MNC have each been studied independently, limited research has explored the relationship between these two constructs within the context of the Saudi healthcare system. Most prior studies have focused either on predictors of job satisfaction or the frequency of missed nursing care without examining their interdependence. Furthermore, differences in healthcare infrastructure, nurse staffing policies, and cultural factors in Saudi Arabia may influence this relationship in unique ways. This study contributes to existing literature by assessing both the prevalence of missed nursing care and job satisfaction levels, and by exploring their association across a diverse sample of nurses working in government hospitals. The findings offer evidence that may guide hospital administrators and policymakers in developing strategies to improve nurse retention, reduce care omissions, and enhance patient safety. Therefore, the aim of this study was to explore the association between missed nursing care and job satisfaction among nurses working in Saudi Arabian government hospitals. The study seeks to address the following objectives:

(1) To assess the prevalence of MNC;
(2) To identify the reasons contributing to MNC;
(3) To evaluate the level of job satisfaction among nurses;
(4) To explore differences in missed nursing care and job satisfaction according to participant demographic and work-related characteristics;
(5) To explore the association between MNC and job satisfaction.

## 2. Materials and Methods

### 2.1. Study Design and Setting

A correlational, cross-sectional study design was employed to investigate the association between missed nursing care and job satisfaction among nurses working in government hospitals in Saudi Arabia. This design was appropriate for examining the relationship between variables and assessing the prevalence of missed nursing care and levels of job satisfaction at a single point in time. Data were collected between February and May 2025. This manuscript was prepared in accordance with the STROBE (Strengthening the Reporting of Observational Studies in Epidemiology) guidelines for cross-sectional studies [21] (see Supplementary Table S1).

The study was conducted exclusively in government hospitals across three major cities: Albahah, Hail, and Almadina. The participating hospitals included King Fahad Hospital in Albahah; King Salman Specialist Hospital, King Khaled Hospital, and Hail General Hospital in Hail; and Ohud Hospital, King Fahad Hospital, and Al-Madinah Al-Munawarah Hospital in Almadina. These institutions represent a range of large tertiary care centers and general hospitals, providing comprehensive healthcare services to diverse patient populations.

### 2.2. Study Population and Sampling

The study population included registered nurses with at least six months of clinical experience in their current hospital setting. Nurses on extended leave, those holding non-clinical administrative positions, nursing interns, student nurses, and nursing trainees were excluded to ensure the inclusion of only fully qualified clinical nursing staff. A convenience sampling technique was used to recruit participants. This approach facilitated access to a diverse group of nurses working in different healthcare settings and cities across Saudi Arabia.

The required sample size was calculated using Raosoft’s online sample size calculator (http://www.raosoft.com/samplesize.html (accessed on 10 January 2025)), based on an estimated nursing population of approximately 2000 nurses across the participating hospitals in the three cities of Hail, Almadina, and Albahah. The calculation applied a 95% confidence level, a 5% margin of error, and an assumed response distribution of 50% to ensure maximum variability. The minimum recommended sample size was determined to be 322 participants.

### 2.3. Data Collection Tools

Data were collected through a structured self-administered questionnaire consisting of three sections:

#### 2.3.1. Demographic and Work-Related Data

This section gathered information on the nurses’ age, gender, marital status, nationality, educational level, years of experience, hospital type, department, shift type, and nurse-to-patient ratio.

#### 2.3.2. Missed Nursing Care (MNC) Scale

The Missed Nursing Care Scale, developed by Kalisch et al. [2], was used to assess both the frequency of missed nursing care and the reasons contributing to care omissions. The instrument consists of two integrated sections. The first section comprises 25 items designed to measure the frequency of missed care activities. Each item is rated on a 5-point Likert scale ranging from 1 (never missed) to 5 (always missed). For analysis purposes, responses were dichotomized, with ratings of 1 to 2 classified as care not missed, and ratings of 3 to 5 considered as care missed, thereby facilitating the calculation of missed care prevalence.

The second section consists of 22 items aimed at evaluating the potential reasons for missed nursing care. These items are rated on a scale from 1 (not a reason) to 4 (significant reason), allowing an assessment of contributing factors such as staffing, communication, and resource availability. The instrument has consistently demonstrated strong psychometric properties, including excellent internal consistency with a reported Cronbach’s alpha of 0.91 in previous nursing research [5]. Test–retest reliability was strong (r ≈ 0.87) for both parts [22]. Subsequent reviews confirm its broad validation: for example, Palese et al. note the MISSCARE survey is “the most widely used tool validated across countries to date” [23].

#### 2.3.3. Job Satisfaction Scale

The Minnesota Satisfaction Questionnaire (MSQ) short form was used to measure overall job satisfaction [14]. The scale consists of 20 items rated on a 5-point Likert scale (1 = very dissatisfied to 5 = very satisfied). Scores range from 20 to 100, with values categorized as low (20–47), moderate (48–76), or high (77–100) job satisfaction. The MSQ has been validated in nursing populations with a Cronbach’s alpha of 0.90 [24]. This scale has also demonstrated adequate test–retest reliability (0.70–0.89) and factorial validity [14]. While formal psychometric validation of the MSQ among nurses in Saudi Arabia has not been published, the scale has been applied in previous Saudi nursing studies (e.g., Al-Haroon & Al-Qahtani [13]; Alotaibi et al. [25]) supporting its contextual relevance.

### 2.4. Data Collection Procedure

Data collection occurred over a six-week period. The electronic survey was designed and uploaded using Google Forms. To maximize reach and ensure that all eligible nurses had the opportunity to participate, the survey link was distributed to the head nurses of each participating hospital across the four cities. The head nurses were responsible for sharing the survey with their respective nursing staff, thereby facilitating access across a diverse range of departments and shift patterns. Prior to participation, all potential participants were provided with written information explaining the study’s objectives, voluntary nature, data confidentiality, and anonymity of responses. Completion and submission of the online survey were considered as providing implied informed consent to participate. The questionnaires were administered in English, as it is the official language of clinical communication, documentation, and professional training in Saudi hospitals. No translation was required. Participants were informed of their right to decline participation or withdraw from the study at any point without penalty.

### 2.5. Ethical Considerations

Ethical approval was obtained from the Research Ethics Committee of the University of Hail (Approval No.: H-2025-625; Date of Approval: 17 February 2025). The study was conducted in accordance with the ethical principles of the Declaration of Helsinki (1975), as revised in 2013. The study adhered to all applicable national and institutional ethical standards. Permission to use the MISSCARE Survey was obtained from the original developer. Use of the MSQ was authorized by the Vocational Psychology Research unit at the University of Minnesota, which holds the copyright. Participants were fully informed about the purpose of the research, the voluntary nature of participation, data confidentiality, and their right to withdraw at any time without penalty. Data were collected anonymously to protect participant privacy and ensure confidentiality.

### 2.6. Statistical Analysis

Data were analyzed using IBM SPSS Statistics version 27.0 (IBM Corp., Armonk, NY, USA). Descriptive statistics were used to summarize demographic and study variables. Categorical data were presented as frequencies and percentages, while continuous variables were reported as medians and interquartile ranges (IQRs) due to non-normal distributions confirmed by the Shapiro–Wilk test. Non-parametric tests were used: the Mann–Whitney U test for two-group comparisons, and the Kruskal–Wallis test for comparisons across three or more groups. Spearman’s rank correlation coefficient was calculated to explore relationships between missed nursing care and job satisfaction. A *p*-value < 0.05 was considered statistically significant for all analyses.

## 3. Results

### 3.1. Sociodemographic Characteristics of the Participants

A total of 366 nurses participated in the study. As summarized in Table 1, the majority were female (87.4%) and over 30 years of age (73.2%). Most were Saudi nationals (96.7%) and held a bachelor’s degree (59.6%). A wide range of departments and shift types were represented, with day shifts being the most common (55.7%). Nurse-to-patient ratios varied, with nearly one-third (32.8%) responsible for 10 or more patients per shift.

The mean scores and standard deviations (SD) for the 25 missed nursing care items reported by the nursing staff reveal notable variation in the frequency of missed care activities across different aspects of clinical practice. The highest reported mean scores, indicative of more frequently missed care, were observed for mouth care (M = 2.99, SD = 1.41), setting up meals for patients who feed themselves (M = 2.92, SD = 1.47), patient bathing and skin care (M = 2.89, SD = 1.42), and feeding the patient while the food is still warm (M = 2.86, SD = 1.47). These findings suggest that essential yet time-consuming fundamental care tasks, particularly those related to personal hygiene, nutrition, and comfort, are the most vulnerable to omission in daily nursing practice (see Supplementary Table S2).

Conversely, the activities with the lowest reported mean scores, indicating comparatively lower frequencies of missed care, included vital signs assessed as ordered (M = 2.17, SD = 1.37), bedside glucose monitoring as ordered (M = 2.25, SD = 1.46), and full documentation of all necessary data (M = 2.26, SD = 1.48). These tasks may be more systematically integrated into clinical workflows and subject to closer monitoring and documentation requirements.

### 3.2. Reasons for Missed Nursing Care

The reasons contributing to missed nursing care as reported by participants are presented in Table 2. The most prominent factor identified was an inadequate number of staff (M = 3.5, SD = 0.9), reflecting a strong perception that staffing shortages frequently lead to care omissions. Other notable contributors included unexpected increases in patient volume or acuity (M = 3.3, SD = 1.0), an inadequate number of assistive or clerical personnel, such as nursing assistants or unit secretaries (M = 3.3, SD = 1.0), and heavy admission and discharge activity (M = 3.3, SD = 1.0).

Additional factors with mean scores of 3.1 or higher included lack of backup support from team members, emotional or physical exhaustion, inadequate support from leadership, and communication breakdowns both within the nursing team and with ancillary departments. The lowest reported factor was inadequate hand-off from the previous shift (M = 2.9, SD = 1.0), suggesting it was perceived as a less frequent cause of missed care in this study (see Supplementary Table S3).

### 3.3. Distribution of Job Satisfaction Levels Among Participants

The median total job satisfaction score for the sample was 60 (interquartile range [IQR]: 44–71; minimum: 4, maximum: 100). Job satisfaction levels were categorized based on total MSQ scores into low (20–47), moderate (48–76), and high (77–100) satisfaction groups. Among the 366 nurses, 25.1% (*n* = 92) reported low job satisfaction, 51.4% (*n* = 188) reported moderate satisfaction, and 23.5% (*n* = 86) reported high satisfaction. These findings suggest that the majority of participants experienced moderate job satisfaction (see Table 2).

### 3.4. Differences in Missed Nursing Care and Job Satisfaction by Participant Characteristics

Table 3 presents the differences in missed nursing care and job satisfaction mean rank scores across participant characteristics. None of the demographic or work-related factors were significantly associated with either missed nursing care or job satisfaction.

For job satisfaction, pediatric nurses (mean rank = 103.03) and those in “other” departments (mean rank = 110.46) demonstrated higher mean rank scores, while the lowest scores were observed among nurses working in obstetrics and gynecology (mean rank = 66.67) and medical–surgical units (mean rank = 73.88). Although these differences were apparent, they did not reach statistically significant levels.

### 3.5. Correlation Between Missed Nursing Care and Job Satisfaction

A statistically significant negative correlation was observed between missed nursing care and job satisfaction (r = −0.267, *p* < 0.001), indicating that higher levels of missed nursing care were associated with lower levels of job satisfaction among the participating nurses (see Table 4).

**Figure 1:**
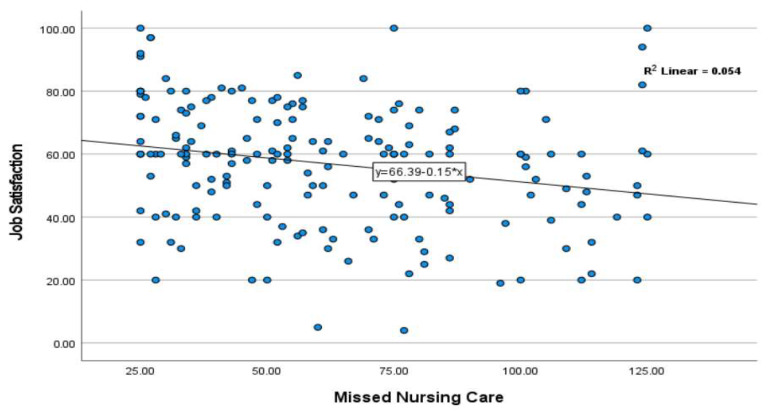
Scatter plot illustrating the relationship between missed nursing care and job satisfaction.

A negative linear trend was also illustrated in the scatter plot, indicating that higher missed nursing care scores were associated with lower job satisfaction levels among nurses (Figure 1).

## 4. Discussion

This study found that nurses in government hospitals across Albahah, Hail, and Almadina reported moderate job satisfaction overall (median MSQ = 60, IQR 44–71), with about one-quarter (25.1%) scoring low, half (51.4%) moderate, and the rest (23.5%) high. These levels align with previous Saudi studies that similarly reported generally moderate satisfaction among nurses [13]. For example, Al-Haroon and Al-Qahtani [13] found roughly half of nurses were satisfied in a major Saudi hospital, with only about one-fifth dissatisfied.

In our sample, basic nursing tasks such as mouth care, bathing, and meal assistance were the most commonly omitted activities, and staffing issues (insufficient personnel and high patient loads) were most often cited as causes of missed care. This pattern is consistent with international surveys, which frequently identify basic care activities (e.g., hygiene and ambulation) as the tasks most often missed [26], and report inadequate staffing as the leading reason for care omissions [6].

A significant negative correlation was found between missed nursing care and job satisfaction, indicating that higher levels of care omissions are associated with lower nurse satisfaction. This finding is consistent with a meta-analysis [4], and with studies conducted in Saudi settings showing a similar association [6]. These concordant results suggest that the current study’s findings are robust; they reinforce the “Missed Care Model” hypothesis that uncompleted care (often due to resource constraints) undermines nurses’ work experience and satisfaction [4,7].

These results have important implications for nursing workforce management and patient care in Saudi Arabia. Staffing adequacy and workload management are key concerns: our nurses cited personnel shortages [10,26] and high patient loads as primary barriers to providing complete care, echoing both international studies and regional findings that missed care in Saudi settings is often driven by labor shortages [6,7].

Because missed care is linked to lower quality and safety of patient care, the finding that higher missed care coexists with lower job satisfaction is troubling. In Thailand, a large survey found that missed care was associated with poorer perceived quality and more adverse events [9]. Likewise, missed nursing care in Saudi hospitals likely undermines patient outcomes and satisfaction. Conversely, improving staffing and work conditions could both reduce missed care and boost nurse morale. Addressing work environment factors (such as clear role allocation, elimination of non-nursing tasks, and supportive supervision) has been shown to decrease missed care and increase satisfaction [9]. In the Saudi healthcare system, this might involve enhancing nurse–patient staffing ratios in government hospitals, accelerating Saudization and local workforce development (so that nurses’ language and cultural alignment improves work conditions), and providing better recognition and career development. Prior Saudi work has emphasized that improving supervision, reward systems, and intrinsic job factors (meaningful work, skill utilization) can raise nurses’ satisfaction [13,27], which in turn may reduce turnover and absenteeism.

### 4.1. Limitations

Several limitations must be acknowledged. The cross-sectional design limits the ability to establish causality; it cannot be determined whether increased missed care causes dissatisfaction or vice versa. Additionally, the use of convenience sampling and the restriction of the study to government hospitals may limit generalizability to the broader nursing population in Saudi Arabia. The sample predominantly comprised Saudi female nurses (96.7%), which may limit the generalizability of the findings. Given that Saudi Arabia employs a substantial proportion of expatriate nurses from diverse backgrounds [28], the results mainly reflect the perspectives of Saudi female nurses and may not represent the broader nursing workforce. Self-reported data introduce the possibility of reporting or recall bias, and cultural factors may influence how nurses perceive and report missed care and satisfaction. Additionally, although the study included nurses from a wide range of clinical departments—such as emergency, intensive care, pediatric, and medical–surgical units—no stratified subgroup analysis was performed. While this allowed for a broad representation of nursing practice, it may have obscured department-specific patterns of missed care. Furthermore, the diversity in workload, patient acuity, and clinical routines across departments could influence the frequency and types of care omitted. Although no statistically significant differences were observed between departments in our sample (see Table 3), this remains a potential limitation. Future studies should consider department-specific analyses to better understand the contextual nature of missed nursing care.

Future research should consider longitudinal or interventional designs to better understand causality between work conditions, missed care, and job satisfaction. Expanding research to include private and military hospitals, as well as additional regions, would provide a more comprehensive understanding. Qualitative studies exploring nurses’ experiences and perceptions of missed care and job satisfaction could further enrich the current understanding. Studies examining the direct patient outcomes associated with reported missed care would provide valuable evidence to inform policy and practice changes.

### 4.2. Perspective for Clinical Practice

The relationship between missed nursing care and job satisfaction observed in this study carries critical implications for clinical practice. Not completing essential nursing tasks is far from a trivial issue—it has been linked to real patient harm. Research indicates that when required care is omitted or delayed, adverse events tend to rise: examples include higher rates of medication errors, patient falls, infections, pressure ulcers, prolonged hospital stays, and even increased mortality [29,30]. Our findings, which show higher missed care correlating with lower nurse satisfaction, are therefore doubly concerning. This pattern suggests that not only are nurses demoralized in such environments, but patient safety and care quality may be compromised simultaneously. Missed care can also erode the work environment by increasing workplace stress and conflict, which in turn fuels burnout and nurses’ intent to leave the profession [31]. The findings underscore that improving nurses’ working conditions and reducing care omissions is not just about nurse welfare—it is a patient safety imperative.

To address these issues, adequate nurse staffing and resource allocation must be top priorities. Both international and local evidence support bolstering staffing levels. For example, a multi-hospital study in Thailand found that poor nurse staffing was associated with more missed care, and in turn, the presence of missed care significantly increased the odds of adverse events and perceived low quality of care [9]. The authors of that study explicitly recommend improving staffing and ensuring sufficient resources as a strategy to reduce missed care and improve patient outcomes [32,33]. Similarly, recent evidence from a neonatal ICU in Saudi Arabia’s Eastern Region showed that missed nursing care was closely tied to overwork and nursing shortages, with detrimental effects on care quality [7]. The study concluded that improving working conditions, nurse staffing, and patient assignment planning should be prioritized to combat missed care in such settings. These findings align with the broader consensus that appropriate staffing is beneficial not only for patients but also for nurses—adequate nurse-to-patient ratios have been shown to improve patient outcomes and lead to greater satisfaction for both patients and nursing staff. From a clinical leadership perspective, this means hospital administrators and policymakers should invest in hiring and retaining an optimal number of nurses, deploying them in a way that balances workloads, and securing the material resources needed so that nurses can complete all required care.

Beyond sheer numbers of staff, the quality of the work environment and team processes plays a pivotal role in ensuring that essential care is not missed. Studies have noted that when hospital units foster a strong safety culture, open communication, and mutual support among healthcare team members, nurses are better able to manage their workload and are less likely to omit care [34]. Nurse managers should encourage a teamwork approach where colleagues help each other and collectively prioritize patient needs during busy shifts—such cooperative problem-solving and “cross-monitoring” has been shown to mitigate missed care even under resource constraints. Additionally, improving the work environment by streamlining workflows and removing unnecessary non-clinical duties from nurses can free up time for patient care. A recent study from Iran urged healthcare leaders to tackle missed care in the context of nursing shortages by enhancing nurses’ work conditions (e.g., manageable workloads, supportive supervision), expanding recruitment and retention efforts, offering competitive compensation, and delegating or reducing non-nursing tasks that burden nurses [31]. Many of these strategies are applicable to the Saudi context as well—for instance, accelerating the development of the local nursing workforce and implementing incentive programs could help alleviate staffing gaps while boosting morale [31].

Finally, an important practical step is to treat missed nursing care as a key quality indicator and actively monitor it. Nurse executives and quality improvement departments should consider regular audits or staff self-reports to identify which care activities are being left undone and why [35]. Proactively tracking missed care trends can alert leadership to problem areas (such as consistently understaffed shifts or units with high workload acuity) and prompt timely corrective actions. In combination, these measures—improving staffing levels, investing in better work environments, fostering teamwork, and monitoring care omissions—create a comprehensive approach to enhance clinical practice. By implementing such changes, healthcare organizations can not only reduce the prevalence of missed nursing care but also improve nurse job satisfaction and retention. In the long run, these improvements translate into safer patient care, higher quality outcomes, and a more resilient nursing workforce, thereby addressing both the human and clinical dimensions of the issue highlighted by our study [9].

## 5. Conclusions

This study provides new evidence on the relationship between MNC and job satisfaction among nurses in Saudi government hospitals. A moderate level of job satisfaction was reported, and fundamental nursing care tasks were frequently missed, often due to staffing constraints and high workloads. The observed negative correlation between missed nursing care and job satisfaction highlights the importance of improving work environments and staffing models to enhance both patient care quality and nurse well-being. By addressing these systemic challenges, healthcare administrators and policymakers can improve nurse retention, reduce care omissions, and ultimately strengthen the quality of patient care. These findings contribute to the international literature by offering insights specific to the Saudi healthcare context, reinforcing global concerns regarding nursing workforce management and care quality.

## Figures and Tables

**Table 1:** Sociodemographic characteristics of the study participants.

Variable	*n* (%)
Age	
≤30 years	98 (26.8)
>30 years	268 (73.2)
Gender	
Male	46 (12.6)
Female	320 (87.4)
Marital Status	
Single	130 (35.5)
Married	206 (56.3)
Divorced/Widowed	30 (8.2)
Nationality	
Saudi	354 (96.7)
Non-Saudi	12 (3.3)
Educational Level	
Diploma	96 (26.2)
Bachelor’s degree	218 (59.6)
Postgraduate degree	52 (14.2)
Years of Experience	
≤6 years	46 (53.5)
>6 years	40 (46.5)
Department	
Emergency room	76 (20.8)
Intensive care unit	44 (12.0)
Medical–surgical unit	40 (10.9)
Pediatric	60 (16.4)
Obstetrics/gynecology	12 (3.3)
Psychiatric unit	12 (3.3)
Others	122 (33.4)
Shift Type	
Day shift	204 (55.7)
Night shift	22 (6.0)
Rotating shifts	140 (38.3)
Nurse-to-Patient Ratio	
1–3 patients	112 (30.6)
4–6 patients	92 (25.1)
7–9 patients	42 (11.5)
10 or more patients	120 (32.8)

**Table 2:** Levels of job satisfaction among nurses.

		*n*	(%)
Job Satisfaction	Low (20–47)	92	(25.1)
	Moderate (48–76)	188	(51.4)
	High (77–100)	86	(23.5)

**Table 3:** Differences in missed nursing care and job satisfaction by participant characteristics.

	Variable	N	Missed Nursing Care		Job Satisfaction	
			Mean Rank	*p*-Value	Mean Rank	*p*-Value
Age	≤30 Years	98	90.59	0.828	91.52	0.941
	>30 Years	268	92.51		92.18	
Gender	Male	46	92.39	0.970	91.43	0.956
	Female	320	91.94		92.08	
Nationality	Saudi	354	92.45	0.531	92.01	0.984
	Non-Saudi	12	78.67		91.58	
Experience	≤6 Years	46	21.41	0.742	23.85	0.299
	>6 Years	40	22.68		19.88	
Marital Status	Single	130	90.14	0.594	93.29	0.902
	Married	206	94.81		90.58	
	Divorced/Widowed	30	80.80		96.17	
Educational level	Diploma	96	82.73	0.273	99.36	0.460
	Bachelor	218	93.57		88.18	
	Postgraduate	52	102.54		94.40	
Department	ER	76	90.76	0.747	87.47	0.099
	ICU	44	100.07		88.80	
	Medical–Surgical Unit	40	94.33		73.88	
	Pediatric	60	87.57		103.03	
	Obstetrics/Gynecology	12	130.58		66.67	
	Psychiatric Unit	12	70.42		78.83	
	Others	122	89.93		110.46	
Shift Type	Day shift	204	95.90	0.226	91.86	0.992
	Night shift	22	106.55		90.36	
	Rotating shifts	140	84.03		92.46	
Nurse-to-Patient Ratio	1–3	112	98.50	0.576	91.87	0.887
	4–6	92	94.38		96.10	
	7–9	42	83.76		85.10	
	10 or more	120	86.99		91.40	

N = Number, ER = Emergency department, ICU = Intensive care unit.

**Table 4:** Correlation between MNC and job satisfaction (N = 366).

Variable	Correlation Coefficient (r)	*p*-Value
Missed Nursing Care vs. Job Satisfaction	−0.267 **	<0.001

Note: ** indicates correlation is statistically significant at the *p* < 0.01 level.

## Data Availability

The data presented in this study are available on reasonable request from the corresponding author. The data are not publicly available due to privacy and ethical restrictions.

## Supplementary Materials

The following supporting information can be downloaded at: https://www.mdpi.com/article/10.3390/nursrep15080296/s1, The following Supplementary Materials are provided with this study: Supplementary Table S1: Frequency of Missed Nursing Care Activities; Supplementary Table S2: Mean Scores of Reasons for Missed Nursing Care; Supplementary Table S3: STROBE Checklist for Cross-Sectional Studies.

## Public Involvement Statement

No public involvement in any aspect of this research.

## Guidelines and Standards Statement

This manuscript was prepared in accordance with the STROBE (Strengthening the Reporting of Observational Studies in Epidemiology) guidelines for cross-sectional studies (von Elm, E. et al., 2007) [21] (see Supplementary Table S3).

## Abbreviations

The following abbreviations are used in this manuscript:

MNC	Missed Nursing Care
ER	Emergency Room
STROBE	Strengthening the Reporting of Observational Studies in Epidemiology
MSQ	Minnesota Satisfaction Questionnaire
ICU	Intensive Care Unit
SPSS	Statistical Package for the Social Sciences
SD	Standard deviation
IQR	Interquartile Range
